# Evaluation of Second-Line Anti-VEGF after First-Line Anti-EGFR Based Therapy in RAS Wild-Type Metastatic Colorectal Cancer: The Multicenter “SLAVE” Study

**DOI:** 10.3390/cancers12051259

**Published:** 2020-05-16

**Authors:** Alessandro Parisi, Alessio Cortellini, Katia Cannita, Olga Venditti, Floriana Camarda, Maria Alessandra Calegari, Lisa Salvatore, Giampaolo Tortora, Daniele Rossini, Marco Maria Germani, Alessandra Boccaccino, Emanuela Dell’Aquila, Claudia Fulgenzi, Daniele Santini, Michele De Tursi, Nicola Tinari, Pietro Di Marino, Pasquale Lombardi, Susana Roselló Keränen, Marisol Huerta Álvaro, Ina Valeria Zurlo, Domenico Cristiano Corsi, Alessandra Emiliani, Nicoletta Zanaletti, Teresa Troiani, Pasquale Vitale, Riccardo Giampieri, Filippo Merloni, Mario Alberto Occhipinti, Paolo Marchetti, Michela Roberto, Federica Mazzuca, Michele Ghidini, Alice Indini, Ingrid Garajova, Federica Zoratto, Simona Delle Monache, Giampiero Porzio, Corrado Ficorella

**Affiliations:** 1 Medical Oncology, St. Salvatore Hospital, University of L’Aquila, 67100 L’Aquila, Italy; k.cannita@gmail.com (K.C.); olgavenditti@yahoo.it (O.V.); porzio.giampiero@gmail.com (G.P.); corrado.ficorella@univaq.it (C.F.); 2 Department of Biotechnology and Applied Clinical Sciences, University of L’Aquila, 67100 L’Aquila, Italy; alessiocortellini@gmail.com; 3 Università Cattolica del Sacro Cuore, 00168 Roma, Italy; florianacamarda1@gmail.com (F.C.); mariaalessandra.calegari@gmail.com (M.A.C.); lisa.salvatore@policlinicogemelli.it (L.S.); giampaolo.tortora@policlinicogemelli.it (G.T.); valeriazurlo26@gmail.com (I.V.Z.); 4 IRCCS-Comprehensive Cancer Center, Policlinico Universitario Agostino Gemelli, 00168 Roma, Italy; 5 Department of Oncology, University Hospital of Pisa, 56100 Pisa, Italy; danielerossini87@gmail.com (D.R.); m.germani93@gmail.com (M.M.G.); boccaccina@gmail.com (A.B.); 6 Department of Translational Research and New Technologies in Medicine, University of Pisa, 56100 Pisa, Italy; 7 Medical Oncology, Campus Bio-Medico, University of Rome, 00128 Rome, Italy; e.dellaquila@unicampus.it (E.D.); c.fulgenzi@unicampus.it (C.F.); d.santini@unicampus.it (D.S.); 8 Department of Medical, Oral and Biotechnological Sciences and Center for Advance Studies and Technology (CAST), G. D’Annunzio University, 66100 Chieti, Italy; detursi@unich.it (M.D.T.); ntinari@unich.it (N.T.); 9 Clinical Oncology Unit, S.S. Annunziata Hospital, 66100 Chieti, Italy; pietrodimarino@gmail.com; 10 Department of Oncology, University of Turin; Candiolo Cancer Institute-FPO-IRCCS, 10060 Candiolo, Italy; pasquale.lombardi@ircc.it; 11 Department of Medical Oncology, INCLIVA Biomedical Research Institute, University of Valencia, 46010 Valencia, Spain; susanark@hotmail.com (S.R.K.); huerta_sol@hotmail.com (M.H.Á.); 12 Instituto de Salud Carlos III, CIBERONC, 28220 Madrid, Spain; 13 UOC Oncologia Medica San Giovanni Calibita Fatebenefratelli Roma, 00186 Roma, Italy; domenicocristiano.corsi@fbf-isola.it (D.C.C.); alessandra.emiliani1@gmail.com (A.E.); 14 Department of Precision Medicine, Università della Campania “Luigi Vanvitelli”, 80131 Napoli, Italy; nicolettazanaletti@gmail.com (N.Z.); troiani.teresa@yahoo.it (T.T.); vitale.pasquale.89@gmail.com (P.V.); 15 Clinica Oncologica e Centro Regionale di Genetica Oncologica, Università Politecnica delle Marche, AOU Ospedali Riuniti-Ancona, 60020 Ancona, Italy; riccardo.giampieri81@gmail.com (R.G.); merloni.filippo@gmail.com (F.M.); 16 Medical Oncology, Policlinico Umberto I, 00161 Rome, Italy; marioalberto.occhipinti@gmail.com (M.A.O.); paolo.marchetti@uniroma1.it (P.M.); 17 Department of Clinical and Molecular Medicine, Oncology Unit, Sant’Andrea Hospital, Sapienza University of Rome, 00189 Rome, Italy; michela.roberto@uniroma1.it (M.R.); federica.mazzuca@uniroma1.it (F.M.); 18 Medical Oncology Unit, Fondazione IRCCS Ca’ Granda Ospedale Maggiore Policlinico, 20122 Milano, Italy; micheleghidini@outlook.com (M.G.); alice.indini@policlinico.mi.it (A.I.); 19 Medical Oncology Unit, University Hospital of Parma, Via Gramsci 14, 43126 Parma, Italy; ingegarajova@gmail.com; 20 Medical Oncology, Santa Maria Goretti Hospital, 04100 Latina, Italy; federica.zoratto@gmail.com; 21 Department of Biotechnological and Applied Clinical Sciences, Laboratory of Applied Biology, University of L’Aquila, 67100 L’Aquila, Italy; simona.dellemonache@univaq.it

**Keywords:** *RAS* wild-type mCRC, anti-angiogenics, second-line treatment, Aflibercept, Bevacizumab, Panitumumab, Cetuximab

## Abstract

Background: The optimal anti-angiogenic strategy as second-line treatment in *RAS* wild-type metastatic colorectal cancer (mCRC) treated with anti-EGFR (Epidermal Growth Factor Receptor) based first-line treatment is still debated. Methods: This multicenter, real-world, retrospective study is aimed at evaluating the effectiveness of second-line Bevacizumab- and Aflibercept-based treatments after an anti-EGFR based first-line regimen. Clinical outcomes measured were: objective response rate (ORR), progression free survival (PFS), overall survival (OS) and adverse events (AEs) profiles. Results: From February 2011 to October 2019, 277 consecutive mCRC patients received Bevacizumab-based (228, 82.3%) or Aflibercept-based (49, 17.7%) regimen. No significant difference was found regarding ORR. The median follow-up was 27.7 months (95%CI: 24.7–34.4). Aflibercept-treated group had a significantly shorter PFS compared to Bevacizumab-treated group (5.6 vs. 7.1 months, respectively) (HR = 1.34 (95%CI: 0.95–1.89); *p* = 0.0932). The median OS of the Bevacizumab-treated group and Aflibercept-treated group was 16.2 (95%CI: 15.3–18.1) and 12.7 (95%CI: 8.8–17.5) months, respectively (HR= 1.31 (95%CI: 0.89–1.93) *p* = 0.16). After adjusting for the key covariates (age, gender, performance status, number of metastatic sites and primary tumor side) Bevacizumab-based regimens revealed to be significantly related with a prolonged PFS (HR = 1.44 (95%CI: 1.02–2.03); *p* = 0.0399) compared to Aflibercept-based regimens, but not with a prolonged OS (HR = 1.47 (95%CI: 0.99–2.17); *p* = 0.0503). The incidence of G3/G4 VEGF inhibitors class-specific AEs was 7.5% and 26.5% in the Bevacizumab-treated group and the Aflibercept-treated group, respectively (*p* = 0.0001). Conclusion: Our analysis seems to reveal that Bevacizumab-based regimens have a slightly better PFS and class-specific AEs profile compared to Aflibercept-based regimen as second-line treatment of *RAS* wild-type mCRC patients previously treated with anti-EGFR based treatments. These results have to be taken with caution and no conclusive considerations are allowed.

## 1. Introduction

With the exception of intensive first-line regimens [1,2], it is now been years that the treatment algorithm of metastatic colorectal cancer (mCRC) patients includes a backbone of fluoropyrimidine-based chemotherapy combined with either oxaliplatin or irinotecan for the first-line approach, followed by the alternative regimen for the second-line treatment. EGFR (Epidermal Growth Factor Receptor) antibodies (Panitumumab and Cetuximab) or anti-angiogenic agents (Bevacizumab, Aflibercept, and Ramucirumab) (Vascular endothelial growth factor [VEGF] pathway inhibitors) are added to these backbones across treatment lines, according to the *RAS* genotype [3]. However, the optimal use and sequencing of these agents has yet to be determined [4].

*RAS* wild-type mCRC patients represent about 40–50% of the overall mCRC population [5] and a common first-line treatment strategy for these patients includes the combination of chemotherapy with anti-EGFR agents [6,7,8,9]. A growing amount of evidences, derived from both retrospective and phase I-II prospective studies, highlights the possibility to obtain clinical benefit from continuing EGFR inhibitors after first-line disease progression in a subset of molecularly selected mCRC patients [10]. However, to date, according to ESMO guidelines [11], the recommended second-line options after an anti-EGFR based first-line treatment include both Bevacizumab-based and Aflibercept-based regimens. The efficacy of Bevacizumab in the second-line setting was assessed in two phase III studies (E3200 and ML18147), which respectively analyzed the effect of adding Bevacizumab to FOLFOX in anti-angiogenesis naïve patients previously treated with FOLFIRI [12], and the efficacy of maintaining Bevacizumab across multiple lines of treatment [13]. On the other hand, the efficacy of Aflibercept was assessed in a phase 3 trial (VELOUR), which analyzed the effect of adding Aflibercept to FOLFIRI as a second-line treatment in mCRC patients progressed to an oxaliplatin-containing regimen, including patients who had previously received Bevacizumab [14]. Therefore, the use of Aflibercept in clinical practice is limited to patients previously treated with oxaliplatin and in combination with an irinotecan-containing regimen. To date, no head to head clinical trial compared Bevacizumab and Aflibercept as second-line treatment in *RAS* wild-type mCRC patients.

The present study is aimed at evaluating the effectiveness of second-line Bevacizumab-based and Aflibercept-based treatments after a first-line anti-EGFR based regimen in *RAS* wild-type mCRC patients in a multicenter real-world cohort.

## 2. Materials and Methods

### 2.1. Patient Eligibility

This retrospective analysis evaluated consecutive *RAS* wild-type mCRC patients, treated with either Bevacizumab-based or Aflibercept-based systemic therapy, at medical oncology department of 13 Italian and one Spanish institutions (Table S1), from February 2011 to October 2019. 

Eligibility criteria were: age ≥ 18 years; histologically confirmed diagnosis of CRC; measurable metastatic disease; confirmed *KRAS* (exons 2, 3, 4) and *NRAS* (exons 2, 3, 4) wild-type genotype; having received an anti-EGFR-based (Panitumumab or Cetuximab) first-line treatment (fluoropyrimidines and/or oxaliplatin and/or irinotecan) and an anti-VEGF based (Bevacizumab or Aflibercept) second-line treatment (fluoropyrimidines and/or oxaliplatin and/or irinotecan) at disease progression. All patients alive at the time of data collection provided informed consent to participate to this retrospective observational non-interventional study. The procedures followed were in accordance with the precepts of good clinical practice and the Declaration of Helsinki. The study was approved by the respective local ethical committees on human experimentation of each institution, after previous approval by the coordinating center (University of L’Aquila, Internal Review Board protocol number 55741, approved on 11 October 2019). The datasets used during the present study are available from the corresponding author upon reasonable request.

### 2.2. Study Design

This is a retrospective, multicenter, observational study, aimed at evaluating the effectiveness of second-line treatments according to the anti-angiogenic regimen received (Bevacizumab-based and Aflibercept-based regimens) in consecutive patients.

The measured clinical outcomes were objective response rate (ORR), progression free survival (PFS), overall survival (OS) and cumulative toxicity. Patients were assessed with radiologic imaging according to the local clinical practice of the participating centers; disease responses were evaluated with the RECIST criteria (version 1.1) [15]. ORR was defined as the portion of patients experiencing an objective response (complete response or partial response) as best response, according to RECIST criteria (version 1.1) [15]. PFS was defined as the length of time from the beginning of second-line treatment to disease progression or death resulting from any cause or to the last contact [16]; OS as the length of time between the beginning of second-line treatment to death resulting from any cause or to the last contact [16]. For PFS as well as for OS, patients without events were considered as censored at the time of the last follow-up. The data cut-off period was January 2020.

Considering the possible unbalanced distribution, the influence of large within group variation and the possible interactions, fixed multivariable regression models were used to estimate clinical outcomes (ORR, PFS, and OS) according to the second-line regimen, by using pre-planned adjusting key covariates [17,18,19]. The key covariates were: age (<70 vs. ≥70 years old) [20], gender (male vs. female) [21], Eastern Cooperative Oncology Group—Performance Status (ECOG-PS) (used as a continuous variable), number of metastatic sites (1 vs. ≥2) [22], primary tumor side (right-side [from caecum to transverse colon] vs. left side [from splenic flexure including rectum]) [23].

Cumulative toxicity, defined as the maximum grade of toxicity experienced was registered according to National Cancer Institute Common Terminology Criteria (NCI-CTC) for Adverse Events (AEs) (version 4 up to January 2018, version 5 from January 2018) and grouped according to severity (grade [G] 1–2 and 3–4). Toxicities were summarized and compared among subgroups according to three key subgroups: VEGF inhibitors class-specific AEs (hypertension, arteriovenous thromboembolic events, fistulae, gastrointestinal perforation, proteinuria, bleeding), hematologic AEs (leukopenia, neutropenia, anemia, thrombocytopenia), and non-hematologic AEs (nausea, vomiting, diarrhea, asthenia, anorexia, mucositis, hand-foot syndrome). Only AEs which occurred in more than 5% of patients were included in the safety analysis.

### 2.3. Molecular Profile Assessment

All the molecular analyses were performed according to the local clinical practice of the participating centers. *KRAS, NRAS* and *BRAF* mutational status was assessed with Sanger sequencing, real-time PCR techniques and next-generation sequencing (NGS) (such as: OncoGenBasic-S1 kit, Seqplexing (Valencia, Spain); Pyromark Q96 ID System, Qiagen (Hilden, Germany); EasyPGX and Myriapod Colon Status, Diatech Pharmacogenetics (Jesi, Italy)). MSI (microsatellite instability) status and/or MMR (mismatch repair) proteins expression were assessed with molecular sequencing (Sanger, Real-Time PCR and NGS) and Immunohistochemistry (IHC) (such as: Applied Biosystem 3500 DX genetic analyzer, Thermo Fisher Scientific (Waltham, MA, USA); Ultraview Universal Detection Kit and Ventana platform, Roche Tissue Diagnostics and Ventana Medical Systems (Tucson, AZ, USA)).

### 2.4. Statistical Analysis

Baseline patients’ characteristics were reported with descriptive statistics and compared among subgroups with the Chi-square test. Chi-square test was also used to compare ORR and the incidence of AEs across subgroups. Logistic regression was used for the multivariate analysis of ORR. Median PFS and median OS were evaluated using the Kaplan–Meier method. Median period of follow-up was calculated according to the reverse Kaplan-Meier method. Cox proportional hazards regression was used for the univariate and multivariate analysis of PFS and OS. The alpha level for all analyses was set to *p* < 0.05. Hazard Ratios (HRs) with 95% confidence intervals (CIs) were calculated using the logistic regression model. All statistical analyses were performed using MedCalc Statistical Software version 18.11.3 (MedCalc Software bvba, Ostend, Belgium; http://www.medcalc.org; 2019).

## 3. Results

### 3.1. Patients Characteristics

A total of 277 consecutive *RAS* wild-type mCRC patients were treated with Bevacizumab-based (228, 82.3%) or Aflibercept-based (49, 17.7%) second-line regimens. The median age was 64.5 years (range: 29–84). Patients features (overall and according to subgroups) are summarized in Table 1. A significantly higher rate of primary tumor resection was reported for the Bevacizumab-treated group (78.9%), compared to the Aflibercept-treated group (49%) (*p* < 0.0001). According to the clinical indication of Aflibercept, also the previously received first-line regimens (*p* < 0.0001) and second-line chemotherapy backbone (*p* = 0.0148) were significantly different. 

### 3.2. Clinical Outcomes Analysis

The activity profile for the overall population and according to subgroups is summarized in Table 2. In the overall population the ORR was 25.8%. No significant ORR difference was found between patients who received Bevacizumab-based and Aflibercept-based regimens.

**Figure 1:**
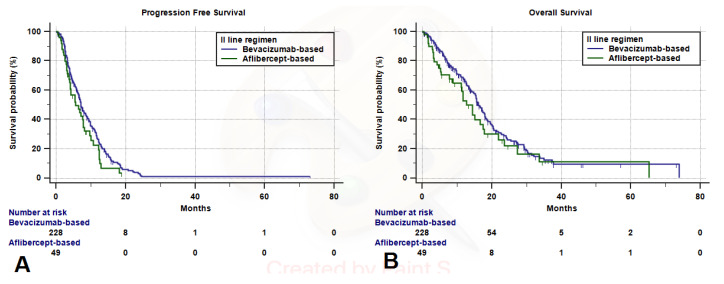
Kaplan–Meyer PFS (**A**) and OS (**B**) curves according to the second-line regimen.

The second-line median follow-up for the study population was 27.7 months (95%CI: 24.7–34.4); median PFS and median OS were 7.1 months (95%CI: 6.3–7.8; 235 progression events) and 15.7 months (95%CI: 14.4–17.4; 94 censored patients). Median PFS of the Bevacizumab-treated group was 7.1 months (95%CI: 6.4–8.5; 195 progression events), while median PFS of the Aflibercept-treated group was 5.6 months (95%CI: 4.1–7.8; 40 progression events), without statistically significant difference at the univariate analysis (HR = 1.34 (95%CI: 0.95–1.89); *p* = 0.0932) (Figure 1A). Median OS of the Bevacizumab-treated group was 16.2 months (95%CI: 15.3–18.1; 77 censored patents), while median OS of the Aflibercept-treated group was 12.7 months (95%CI: 8.8–17.5; 17 censored patients), without statistically significant differences at the univariate analysis (HR = 1.31 (95%CI: 0.89–1.93)]; *p* = 0.1600) (Figure 1B). Table 3 and Table 4 summarized the results of univariate and multivariate analyses of PFS and OS, respectively. After adjusting for the key covariates Bevacizumab-based regimens revealed to be significantly related with a prolonged PFS (HR = 1.44 (95%CI: 1.02–2.03); *p* = 0.0399) compared to Aflibercept-based regimens, but not with a prolonged OS (HR = 1.47 (95%CI: 0.99–2.17); *p* = 0.0503).

### 3.3. Toxicity Analysis

**Figure 2:**
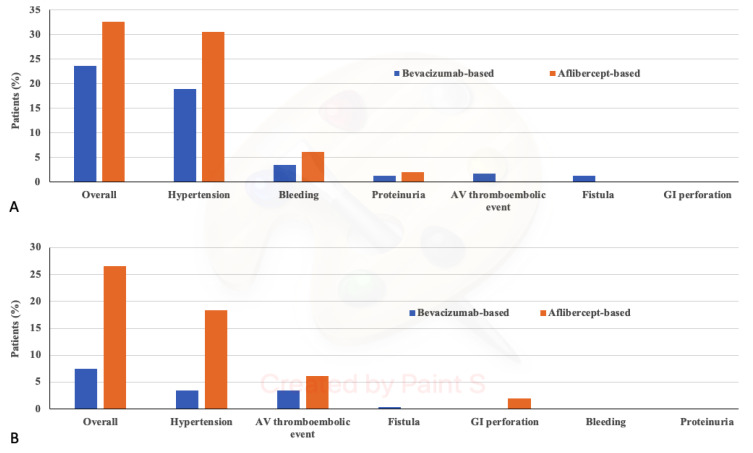
Incidence of G1/G2 (**A**) and G3/G4 (**B**) VEGF inhibitors class-specific adverse events according to the second-line regimen. AV: arteriovenous; GI: gastrointestinal.

The toxicity profile for the overall study population and according to subgroups is summarized in Table 5. The incidence of G1/G2 VEGF inhibitors class-specific AEs was 23.7% and 32.7% in the Bevacizumab-treated group and in the Aflibercept-treated group, respectively (*p* = 0.1908). The incidence of G3/G4 VEGF inhibitors class-specific AEs was 7.5% and 26.5% in the Bevacizumab-treated group and in the Aflibercept-treated group, respectively (*p* = 0.0001) (Figure 2). The incidence of G1/G2 non hematologic AEs was 36.4% and 59.2% in the Bevacizumab-treated group and in the Aflibercept-treated group, respectively (*p* = 0.0033), while the incidence of G3/G4 non hematologic AEs was 4.4% and 10.2%, respectively (*p* = 0.1032). The incidence of G1/G2 hematologic AEs was 24.6% and 22.4% in the Bevacizumab-treated group and in the Aflibercept-treated group, respectively (*p* = 0.7545), while the incidence of G3/G4 hematologic AEs was 3.1% and 18.4%, respectively (*p* < 0.0001).

### 3.4. Maintenance Regimens and Post-Progression Treatments

A total of 67 patients (29.4%) and nine patients (18.4%) underwent a maintenance therapy after an induction phase, in the Bevacizumab-treated group and in the Aflibercept-treated group, respectively (*p* = 0.2236). A total of 136 patients (69.4%) and 24 patients (60%) were treated with a third-line systemic therapy, among those who discontinued second-line treatment in the Bevacizumab-treated group and Aflibercept-treated group, respectively (*p* = 0.5930). Table 6 summarized maintenance treatments characteristics, causes of second-line discontinuation and third-line treatments.

## 4. Discussion

This observational retrospective study intends to provide further data outside the clinical trial framework. To the best of our knowledge this is the first study aimed at comparing the effectiveness of Bevacizumab-based and Aflibercept-based second-line regimens in *RAS* wild-type mCRC patients. Moreover, the phase III E3200, ML18147 and VELOUR studies enrolled patients who had not previously received EGFR inhibitors [12,13,14], therefore, little is known about the clinical outcomes with Bevacizumab and Aflibercept in this setting. 

Findings from preclinical studies showed that acquired resistance to EGFR inhibitors derives from the emergence of novel mutations in the *RAS* protein family and that *KRAS* mutant isoforms could be a VEGF expression inducer, which in turn is targetable by anti-angiogenic treatments [24,25,26,27]. Data from the first-line setting further suggest that an EGFR-based first-line therapy might create a favorable precondition for second-line treatments with VEGF-targeted antibodies [28], particularly in left-sided colon cancer [29]. Regarding the sequential use of Bevacizumab or Aflibercept after an anti-EGFR therapy, three retrospective studies [30,31,32], two of which were conducted only among Asian population, showed that the clinical outcomes of mCRC patients treated with a second-line anti-angiogenic therapy seemed to be comparable with those reported in the phase III studies [12,13,14].

Despite the unbalanced grouping of the study population according to the received regimens (82.3% Bevacizumab-based vs. 17.7% Aflibercept-based), most of the patients characteristics were balanced between the subgroups, such as elderly patients, number of metastatic sites and primary tumor location (see Table 1). On the other hand, there was a statistically significant difference of primary tumor resection rate between Bevacizumab-treated group and Aflibercept-treated group (78.9% vs. 49%, *p* < 0.0001), and this might have affected the clinical outcomes [33]. The clinical indication of Aflibercept (limited to patients previously treated with oxaliplatin and in combination with an irinotecan-containing regimen) explain instead the significant differences according to the previously received first-line regimen and to the second-line chemotherapy backbone. The prevalence of left-sided tumors (74.4%) and the probable attitude not to treat with first-line EGFR-inhibitors *BRAF* mutant patients [34], are aligned to the *BRAF* mutational status (almost 90% of patients were *BRAF* wild-type), identifying a study population with good prognosis overall [35].

Even though studies results comparisons are not methodologically correct, some speculations are allowed. The median PFS of the Bevacizumab-treated group (7.1 months) was comparable to the PFS reported in the E3200 and ML18147 trials (7.3 and 5.7 months, respectively) [12,13], whereas the median PFS of the Aflibercept-treated group (5.6 months) was slightly worse than the PFS reported for the experimental arm of the VELOUR study (6.9 months) [14]. The median OS of the Bevacizumab-treated group (16.2 months) was slightly better than the OS reported in the experimental arms of E3200 and ML18147 trials (12.9 and 11.2 months, respectively) [12,13], while the median OS of the Aflibercept-treatment group (12.7 months) was comparable to the OS of the experimental arm of the VELOUR study (13.5 months) [14]. Additionally, the ORR of Bevacizumab-treated (25.7%) and Aflibercept-treated (26.1%) groups resulted to be higher compared to the experimental arms of the E3200 (23%), the ML18147 (5%) and the VELOUR (19.9%) trials. Surely, in addition to some study populations’ differences, the genotype selection of our cohort (only *RAS* wild-type patients were eligible) might also partially explain these discrepancies. Interestingly, genotype based post-hoc analyses reported an OS of 15.4 months for *KRAS* wild-type patients of the experimental arm of the ML18147 [36], and an OS of 16.0 months for *RAS* wild-type patients of the experimental arm of the VELOUR trial [37]. Moreover, we have to take into account that most of our patients received active third-line regimens, such as Regorafenib and Trifluridine-tipiracil, which might have affected the OS.

Intriguingly, the multivariate analysis revealed that the Aflibercept-treated group had a statistically significant shorter PFS compared to the Bevacizumab-treated group (HR = 1.44 (95%CI: 1.02–2.03); *p* = 0.0399), whereas a not significant trend towards a shorter OS was reported (HR = 1.47 (95%CI: 0.99–2.17); *p* = 0.0503). Concerning safety data, we found a significant higher incidence of G3/G4 VEGF inhibitors class-specific AEs among Aflibercept-treated patients, compared to the Bevacizumab-treated patients (26.5% vs. 7.5%, *p* = 0.0001). This aspect might be also related to the different pharmacodynamic mechanisms of action of Bevacizumab (a monoclonal antibody which targets VEGF-A) and Aflibercept (a fusion protein which targets both VEGF-A, VEGF-B and placental growth factor (PIGF)) [38]. Furthermore, a statistically significant difference in the incidence of G1/G2 non hematologic AEs (36.4% vs. 59.2%, *p* = 0.0033) and G3/G4 hematologic AEs (3.1% vs. 18.4%) to the detriment of the Aflibercept-treated patients, was found. The latter aspect could be related to the different chemotherapy backbone (FOLFIRI in 32% of Bevacizumab-treated group and 100% in the Aflibercept-treated group, *p* = 0.0148).

Our results suggest a slightly better clinical performance for second-line Bevacizumab-based regimens compared to Aflibercept-based regimens. In our opinion, the different safety profile might had affected the effectiveness of Aflibercept-based regimens compared to Bevacizumab-based regimens, leading to a higher discontinuation rate (17.5% vs. 9.2%, respectively) and a worse PFS.

According to the RAISE trial results [39], it would have been interesting to take into consideration Ramucirumab-based second-line regimens, however, Ramucirumab is not reimbursed in Italy as second-line treatment in mCRC patients. 

Results from important prospective phase II-III studies, comparing different sequencing strategies of available biological agents for *RAS* wild-type patients, are awaited. The STRATEGIC-S1 trial (NCT01910610) [40] is an international, open-label, randomized, multicenter phase III trial designed to compare two standard treatment strategies in unresectable *RAS* wild-type mCRC patients: an oxaliplatin-based second-line regimen with Bevacizumab after fist line FOLFIRI-Cetuximab vs. an irinotecan-based second-line regimen with Bevacizumab after a first-line OPTIMOX Bevacizumab, followed by an anti-EGFR based third-line treatment. The DISTINCTIVE trial (NCT04252456) [41] is a prospective phase II trial, designed to evaluate the efficacy of FOLFIRI-Aflibercept as second-line treatment of *RAS* wild-type mCRC patients after an oxaliplatin/fluoropyrimidines-based first-line regimen combined with either Panitumumab or Cetuximab. 

There are some obvious limitations in this study, including its retrospective design, which expose to selection bias, therefore the results must be taken with caution. Further analysis with a larger sample size and a prospective translational design are certainly needed to better define and personalize the anti-angiogenic strategy as a second-line treatment in *RAS* wild-type mCRC patients.

## 5. Conclusions

Our analysis seems to reveal that Bevacizumab-based regimens have a slightly better efficacy and safety profile compared to Aflibercept-based regimens as second-line treatment of *RAS* wild-type mCRC patients who received first-line anti-EGFR based treatments. These results have to be taken with caution and no conclusive consideration are allowed.

## Figures and Tables

**Table 1:** Patient and tumor characteristics in overall, Bevacizumab-based, and Aflibercept-based population.

Characteristic	Overall N (%)	Bevacizumab-Based N (%)	Aflibercept-Based N (%)	
	277 (100)	228 (82.3)	49 (17.7)	*p* Value
**Age** Median (years) Range (years) Elderly (≥70)	64.5 29–84 90 (32.5)	65.5 30–84 76 (33.3)	63 29–81 14 (28.6)	0.5192
**Sex** Male Female	168 (60.6) 109 (39.4)	139 (61.0) 89 (39.0)	29 (59.2) 20 (40.8)	0.8172
**ECOG-PS** 0 1 2	147 (53.1) 116 (41.9) 14 (5.0)	118 (51.7) 100 (43.9) 10 (4.4)	29 (59.2) 16 (32.6) 4 (8.2)	0.6953 #
**N° of metastatic sites** 1 ≥2	93 (33.6) 184 (66.4)	74 (32.5) 154 (67.5)	19 (38.8) 30 (61.2)	0.3963
**Sideness** Right-side Left-side/Rectum	71 (25.6) 206 (74.4)	58 (25.4) 170 (74.6)	13 (26.5) 36 (73.5)	0.8740
**Primary tumor resection** Yes No	204 (73.6) 73 (26.4)	180 (78.9) 48 (21.1)	24 (49.0) 25 (51.0)	<0.0001
***BRAF*** Wild-type V600E mutated Not-V600E mutated NA	249 (89.9) 3 (1.1) 1 (0.4) 24 (8.6)	204 (89.5) 2 (0.9) 1 (0.4) 21 (9.2)	45 (91.8) 1 (2.0) 0 (0.0) 3 (6.2)	0.4027 #
**MMR/MSI** Proficient/wild-type Deficient/mutated NA	96 (34.7) 5 (1.8) 176 (63.5)	80 (35.1) 5 (2.2) 143 (62.7)	16 (32.7) 0 (0) 33 (67.3)	0.6361 #
**I-line treatment** FOLFIRI-Cetuximab FOLFOX-Cetuximab FOLFOX-Panitumumab FOLFIRI-Panitumumab mFOLFOXIRI-anti-EGFR 5-FU/Cape-anti-EGFR	142 (51.3) 19 (6.8) 92 (33.2) 5 (1.8) 11 (4.0) 8 (2.9)	140 (61.4) 15 (6.6) 51 (22.4) 5 (2.2) 9 (3.9) 8 (3.5)	2 (4.1) 4 (8.1) 41 (83.7) 0 (0) 2 (4.1) 0 (0)	<0.0001 #
**II-line chemotherapy backbone** FOLFOX/XELOX FOLFIRI FOLFOXIRI 5-FU/Cape	128 (46.2) 122 (44.1) 2 (0.7) 25 (9.0)	128 (56.1) 73 (32.0) 2 (0.9) 25 (11.0)	0 (0) 49 (100) 0 (0) 0 (0)	0.0148 #

NA: Not available/evaluable; MMR/MSI: Mismatch repair protein/Microsatellite instability; mFOLFOXIRI: modified FOLFOXIRI; 5-FU: 5-Fluorouracil; Cape: Capecitabine. # Chi-square test for trend.

**Table 2:** Univariate and multivariate analysis for objective response rate.

	OBJECTIVE RESPONSE RATE					
	Univariate Analysis			Multivariate Analysis		
Variable (Comparator)	Responses-Ratio	ORR (95% CI)	*p*-Value	Coeff.	St. Err.	*p*-Value
**Overall**	68/264	25.8 (20.0–32.6)	*-*	-	-	*-*
**II Line regimen** Bevacizumab-based Aflibercept-based	56/218 12/46	25.7 (19.4–33.3) 26.1 (13.4–45.6)	0.9553	0.0126	0.3762	0.9733
**ECOG-PS** 0 1 2	39/141 26/111 3/12	27.7 (19.7-37.8) 23.4 (15.3–34.3) 25.0 (5.1–73.1)	0.7458	–0.0996	0.2564	0.6976
**No. of metastatic sites** 1 site ≥2 sites	29/89 39/175	32.6 (21.8–46.8) 22.3 (15.8–30.5)	0.0710	–0.4905	0.3010	0.1032
**Sex** Female Male	30/103 38/161	29.1 (19.6–41.6) 23.6 (16.7–32.4)	0.3177	0.2497	0.2899	0.3891
**Age** Elderly Non-elderly	24/86 44/178	27.9 (17.9–41.5) 24.7 (17.9–33.2)	0.5798	0.0945	0.3219	0.7639
**Sideness** Right-side Left-side	23/66 45/198	34.8 (22.1–52.3) 22.7 (16.6–30.4)	0.0516	0.5516	0.3219	0.0866

**Table 3:** Univariate and multivariate analysis for progression-free survival.

	PROGRESSION FREE SURVIVAL	
	Univariate Analysis	Multivariate Analysis
VARIABLE	HR (95% CI); *p*-Value	HR (95% CI); *p*-Value
**II Line regimen** Aflibercept-based vs. Bevacizumab-based	1.34 (0.95–1.89); *p* = 0.0932	1.44 (1.02–2.03); *p* = 0.0399
**ECOG-PS** Continuous	1.44 (1.15–1.82); *p* = 0.0013	1.36 (1.07–1.72); *p* = 0.0107
**No. of metastatic sites** ≥2 sites vs. 1 site	1.68 (1.27–2.21); *p* = 0.0002	1.56 (1.18–2.08); *p* = 0.0019
**Sex** Female vs. Male	0.92 (0.71–1.20); *p* = 0.5564	0.91 (0.70–1.19); *p* = 0.5184
**Age** Non-elderly vs. Elderly	0.99 (0.75–1.31); *p* = 0.9725	0.94 (0.70–1.26); *p* = 0.6950
**Sideness** Right-side vs. Left-side	0.79 (0.59–1.06); *p* = 0.1224	0.87 (0.64–1.18); *p* = 0.3785

**Table 4:** Univariate and multivariate analysis for overall survival.

	OVERALL SURVIVAL	
	Univariate Analysis	Multivariate Analysis
VARIABLE	HR (95% CI); *p*-Value	HR (95% CI); *p*-Value
**II Line regimen** Aflibercept-based vs. Bevacizumab-based	1.31 (0.89–1.93); *p* = 0.1600	1.47 (0.99–2.17); *p =* 0.0503
**ECOG-PS** Continuous	1.98 (1.53–2.57); *p* < 0.0001	1.81 (1.38–2.37); *p <* 0.0001
**No. of metastatic sites** ≥ 2 sites vs. 1 site	2.17 (1.56–3.03); *p* < 0.0001	1.90 (1.35–2.67); *p =* 0.0002
**Sex** Female vs. Male	0.72 (0.53–0.98); *p* = 0.0390	0.80 (0.59–1.09); *p =* 0.1727
**Age** Non-elderly vs. Elderly	1.10 (0.81–1.48); *p* = 0.5316	0.98 (0.72–1.35); *p =* 0.9411
**Sideness** Right-side vs. Left-side	0.94 (0.68–1.30); *p =* 0.7295	0.99 (0.71–1.38); *p =* 0.9582

**Table 5:** Adverse events in overall, Bevacizumab-based and Aflibercept-based population.

	Overall N (277)		Bevacizumab-Based N (228)		Aflibercept-Based N (49)	
**Adverse Events (AE)**	G1–G2 N (%)	G3–G4 N (%)	G1–G2 N (%)	G3-G4 N (%)	G1–G2 N (%)	G3–G4 N (%)
**VEGF inhibitors class-specific**	70 (25.3)	29 (10.5)	54 (23.7)	17 (7.5)	16 (32.7)	13 (26.5)
Hypertension	58 (82.9)	17 (58.6)	43 (79.6)	8 (47.1)	15 (93.8)	9 (69.2)
AV thromboembolic event	4 (5.7)	11 (37.9)	4 (7.4)	8 (47.1)	0 (0)	3 (23.1)
Bleeding	11 (15.7)	0 (0)	8 (14.8)	0 (0)	3 (18.8)	0 (0)
Fistula	3 (4.3)	1 (3.4)	3 (5.6)	1 (5.9)	0 (0)	0 (0)
GI perforation	0 (0)	1 (3.4)	0 (0)	0 (0)	0 (0)	1 (7.7)
Proteinuria	3 (4.3)	1 (3.4)	3 (5.6)	0 (0)	1 (6.3)	0 (0)
**Hematologic**	67 (29.4)	16 (5.8)	56 (24.6)	7 (3.1)	11 (22.4)	9 (18.4)
Leukopenia	8 (11.9)	3 (18.7)	7 (12.5)	1 (14.3)	1 (11.1)	2 (20)
Neutropenia	37 (55.2)	13 (81.2)	32 (57.1)	5 (71.4)	5 (55.6)	8 (80)
Anemia	47 (70.1)	4 (25.0)	40 (71.4)	3 (42.9)	7 (77.8)	1 (10)
Thrombocytopenia	29 (43.3)	1 (6.2)	21 (37.5)	1 (14.3)	8 (88.9)	0 (0)
**Non hematologic**	112 (40.4)	14 (5.1)	83 (36.4)	10 (4.4)	29 (59.2)	4 (8.2)
Asthenia	46 (41.1)	3 (21.4)	31 (37.3)	2 (12.5)	15 (50.0)	1 (20)
Anorexia	16 (14.3)	0 (0)	10 (12.0)	0 (0)	6 (20)	0 (0)
Diarrhea	60 (53.6)	5 (35.7)	40 (48.2)	3 (25.0)	20 (66.7)	2 (40)
Nausea	33 (29.5)	2 (14.3)	24 (28.9)	2 (25.0)	9 (30.0)	0 (0)
Vomiting	7 (6.2)	1 (7.1)	4 (4.8)	1 (12.5)	3 (10)	0 (0)
Mucositis/stomatitis	33 (29.5)	2 (14.3)	21 (25.3)	1 (12.5)	12 (40)	1 (20)
HFS	9 (8.0)	1 (7.1)	8 (9.6)	1 (12.5)	1 (3.3)	0 (0)

**Table 6:** Second- and third-line treatment characteristics in overall, Bevacizumab-based and Aflibercept-based population.

	Overall Population N (%)	Bevacizumab-Based N (%)	Aflibercept-Based N (%)	*p*-Value
**Characteristic**	277 (100)	228 (82.3)	49 (17.7)	
**II-line maintenance treatment**	76 (27.4)	67 (29.4)	9 (18.4)	0.2236
5-FU/Cape + antiangiogenic	63 (22.7)	56 (24.6)	7 (14.3)	
Antiangiogenic alone	10 (3.6)	8 (3.5)	2 (4.1)	
5-FU/Cape alone	3 (1.1)	3 (1.3)	0 (0)	
**II-line discontinued**	236 (85.2)	196 (86.0)	40 (81.6)	0.8425
**Cause of discontinuation**				
Disease Progression	193 (81.8)	161 (82.1)	32 (80.0)	
Toxicity	25 (10.6)	18 (9.2)	7 (17.5)	
Patient rest/refusal	10 (4.2)	9 (4.6)	1 (2.5)	
Palliative surgery or locoregional treatments	8 (3.4)	8 (4.1)	0 (0)	
**III-line treatment**	160 (67.8) ¥	136 (69.4) ¥	24 (60.0) ¥	0.5930
Regorafenib	57 (35.6)	47 (34.6)	10 (41.7)	
Trifluridine-tipiracil	15 (9.4)	12 (8.8)	3 (12.5)	
Other (CT or Clinical Trial)	48 (30.0)	45 (33.1)	3 (12.5)	
Anti-EGFR retreatment	40 (25.0)	32 (23.5)	8 (33.3)	

NA: Not available/evaluable; mFOLFOXIRI: modified FOLFOXIRI; Cet: Cetuximab; Pani: Panitumumab; 5-FU: 5-Fluorouracil; Cape: Capecitabine; CT: Chemotherapy retreatment; ¥ computed using the number of patients who discontinued II-line as denominator.

## Supplementary Materials

The following are available online at https://www.mdpi.com/2072-6694/12/5/1259/s1, Table S1: List of participating centers.
