# Supplementary material for: Evaluation of Second-Line Anti-VEGF after First-Line Anti-EGFR Based Therapy in RAS Wild-Type Metastatic Colorectal Cancer: The Multicenter “SLAVE” Study
Source: Cancers (Basel). 2020 May 16;12(5):1259. doi: 10.3390/cancers12051259 (PMC7281759; doi:10.3390/cancers12051259)
Supplement: Supplementary file 1 [file cancers-12-01259-s001.pdf]

cancers

Supplementary Materials

Evaluation of Second-line Anti-VEGF after First-line Anti-EGFR Based Therapy in RAS Wild-Type Metastatic Colorectal Cancer: The Multicenter "SLAVE" Study

Table S1. List of participating centres.

Institution	Department
St. Salvatore Hospital, University of L'Aquila, L'Aquila	Medical Oncology
Università Cattolica del Sacro Cuore, Roma. Policlinico Universitario Agostino Gemelli, IRCCS - Comprehensive Cancer Center.	Medical Oncology
Department of Oncology, University Hospital of Pisa, Pisa, Italy and Department of Translational Research and New Technologies in Medicine, University of Pisa, Pisa	Medical Oncology
Campus Bio-Medico University, Rome	Medical Oncology
Department of Medical, Oral & Biotechnological Sciences University G. D'Annunzio, Chieti-Pescara, Italy; Clinical Oncology Unit, S.S. Annunziata Hospital, Chieti	Medical Oncology
Clinica Oncologica e Centro Regionale di Genetica Oncologica, Università Politecnica delle Marche, AOU Ospedali Riuniti-Ancona, Italy	Medical Oncology
UOC Oncologia Medica San Giovanni Calibita Fatebenefratelli Roma	Medical Oncology
Department of Oncology, University of Turin; Candiolo Cancer Institute - FPO-IRCCS, Candiolo - Turin	Medical Oncology
Department of Medical Oncology, INCLIVA Biomedical Research Institute, University of Valencia, 46010 Valencia, Spain	Medical Oncology
Department of Precision Medicine, Università della Campania "Luigi Vanvitelli", 80131 Naples	Medical Oncology
Policlinico Umberto I, Rome	Medical Oncology
St. Andrea Hospital, Rome	Medical Oncology
Fondazione IRCCS Ca' Granda Ospedale Maggiore Policlinico, Milan	Medical Oncology
Medical Oncology Unit, University Hospital of Parma	Medical Oncology
S Maria Goretti Hospital, Latina	Medical Oncology

© 2020 by the authors. Licensee MDPI, Basel, Switzerland. This article is an open access article distributed under the terms and conditions of the Creative Commons Attribution (CC BY) license (http://creativecommons.org/licenses/by/4.0/).
